# Supplementary figures and images for: Mortality rates among COVID-19 patients hospitalised during the first three waves of the epidemic in Milan, Italy: A prospective observational study
Source: PLoS One. 2022 Apr 11;17(4):e0263548. doi: 10.1371/journal.pone.0263548 (PMC9000097; doi:10.1371/journal.pone.0263548)

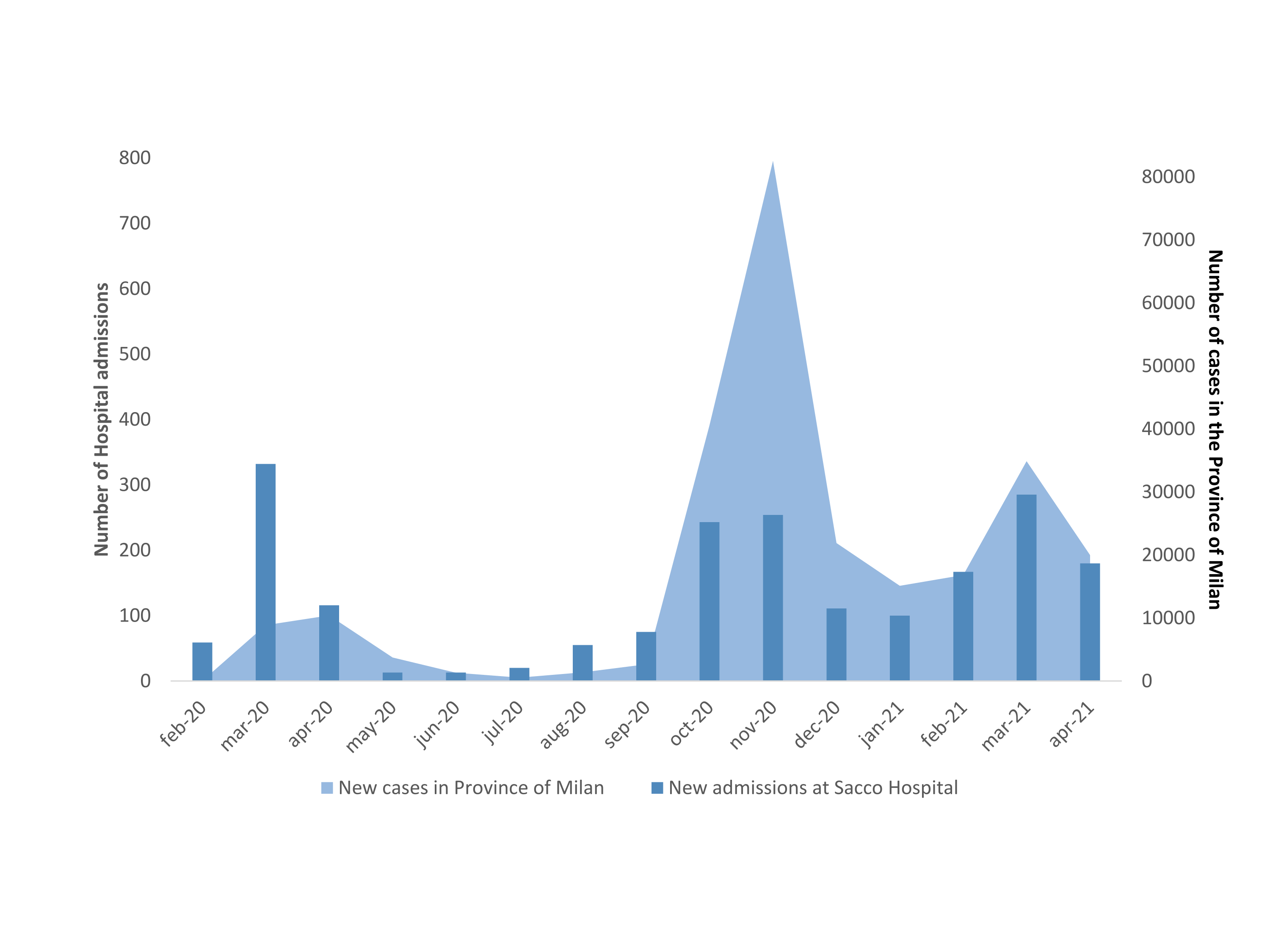

Supplement: S1 Fig — (TIF) [file pone.0263548.s001.tif]

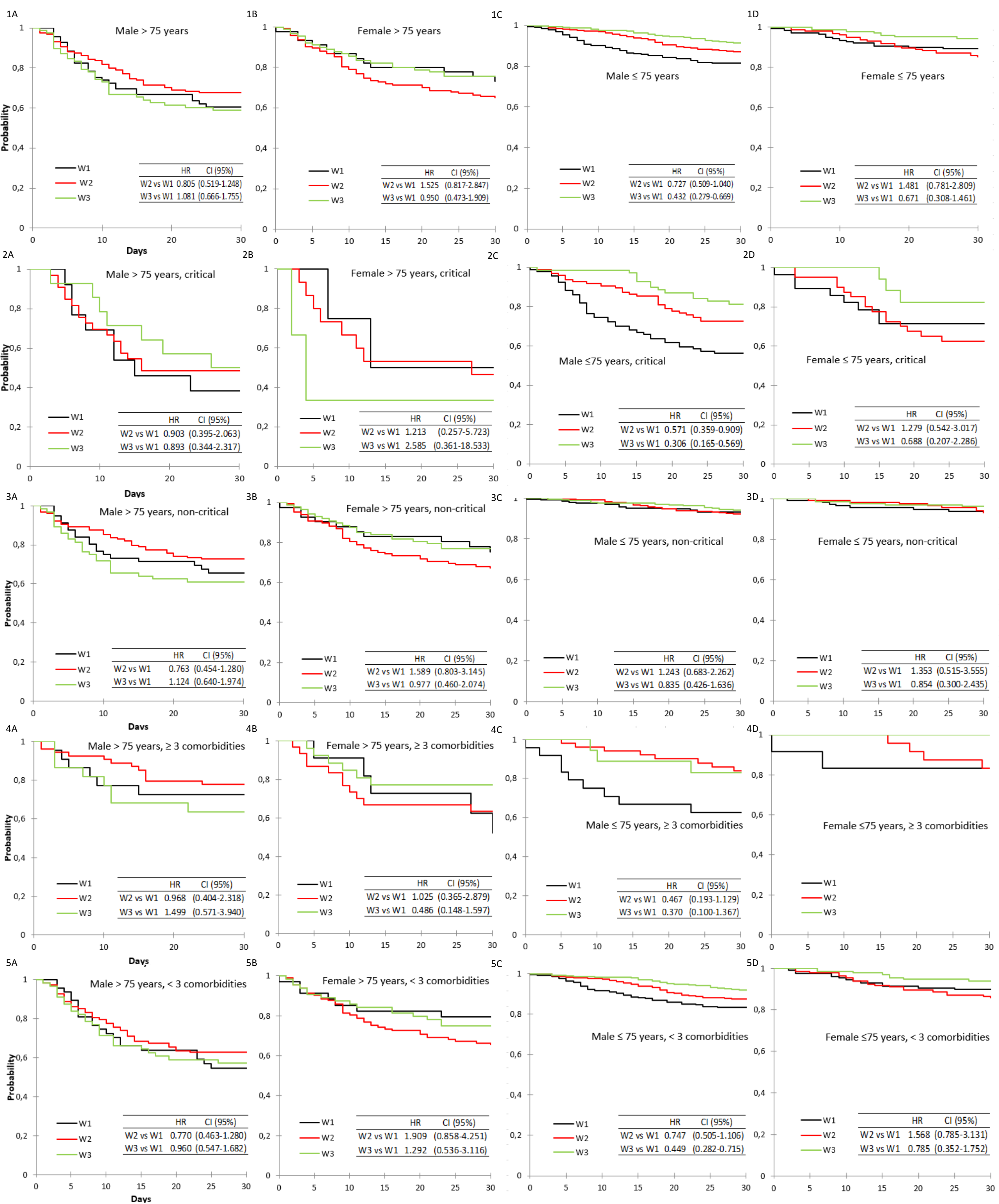

Supplement: S2 Fig — 1) A,B,C,D being male or female and aged >75 or ≤75 years; 2) A,B,C,D being male or female, aged >75 or ≤75 years, and presenting with critical disease upon hospital admission; 3) A,B,C,D being male or female, aged >75 or ≤75 years, and presenting without critical disease upon hospital admission; 4) A,B,C,D being male or female, aged >75 or ≤75 years, and with ≥3 co-morbidities; 5) A,B,C,D being male or female, aged >75 or ≤75 years, and presenting with <3 co-morbidities. (PDF) [file pone.0263548.s002.pdf]
