# Supplementary material for: Mortality rates among COVID-19 patients hospitalised during the first three waves of the epidemic in Milan, Italy: A prospective observational study
Source: PLoS One. 2022 Apr 11;17(4):e0263548. doi: 10.1371/journal.pone.0263548 (PMC9000097; doi:10.1371/journal.pone.0263548)
Supplement: S1 Table — (DOCX) [file pone.0263548.s003.docx]

|  | HR | HR 95% CI |  | AHR | AHR 95% CI |  |
| --- | --- | --- | --- | --- | --- | --- |
| Age (per additional 10 years) | 1.98 | 1.80-2.17 |  | 2.36 | 2.11-2.64 |  |
| Time from symptom onset (per additional day) | 0.99 | 0.97-1.01 |  | 0.99 | 0.97-1.01 |  |
| COVID-19 epidemic wave 3 *vs* wave 2 | 0.64 | 0.51-0.82 |  | 0.85 | 0.65-1.11 |  |
| Monthly cases of SARS-CoV-2 infection  in the province of Milan (per additional 1000)* | 1.01 | 1.01-1.02 |  | 1.00 | 0.00-1.01 |  |
| Male *vs* female | 0.93 | 0.73-1.17 |  | 1.21 | 0.95-1.55 |  |
| Obesity (yes *vs* no) | 1.21 | 0.94-1.54 |  | 1.76 | 1.36-2.29 |  |
| No. of co-morbidities (≥3 *vs* <3) | 1.49 | 1.13-1.96 |  | 0.91 | 0.69-1.21 |  |
| Disease severity (severe *vs* mild/moderate) | 2.32 | 1.75-3.07 |  | 2.31 | 1.72-3.08 |  |
| Disease severity (critical *vs* mild/moderate) | 3.34 | 2.50-4.45 |  | 4.62 | 3.35-6.38 |  |

Supplementary Table 1. Cox model of the factors associated with death during epidemic wave 2 and 3, adjusted for the monthly number of cases of SARS-CoV-2 infection reported in the province of Milan.

HR: hazard ratio; AHR: adjusted hazard ratio; CI: confidence Interval.

* each hospital admission date was attributed with the monthly number of SARS-CoV-2 infections recorded in the province of Milan [22]
